# A Case Report of the Combined Use of Neodymium-Doped Yttrium Aluminum Garnet and Argon Lasers in Treating Luminal Obstruction by Iris Tissue in a Preserflo MicroShunt Implant

**DOI:** 10.7759/cureus.84799

**Published:** 2025-05-25

**Authors:** Thanam Tamil Chelvan, Rupini Yogesvaran, Teck Chee Cheng, Jemaima Che Hamzah

**Affiliations:** 1 Department of Ophthalmology, Hospital Canselor Tuanku Muhriz, Universiti Kebangsaan Malaysia, Kuala Lumpur, MYS

**Keywords:** argon laser, glaucoma, intraocular pressure, minimally invasive glaucoma surgery, neodymium-doped yttrium aluminum garnet laser, preserflo microshunt complications

## Abstract

We aim to report a case of iris tissue obstructing the lumen following rotation of the Preserflo MicroShunt (PMS) and subsequent laser therapy, which restored the shunt's function.

A 65-year-old man with bilateral primary open-angle glaucoma (right eye: early stage, left eye: severe stage) had suboptimal intraocular pressure (IOP) control in his left eye despite maximal medical therapy. Hence, with an uneventful operative course, PMS implantation augmented with mitomycin C (0.04%) was performed in the left eye. However, one week post-surgery, his IOP spiked to 30 mmHg, and anterior segment examination revealed that the adjacent iris tissue obstructed the lumen of the PMS. Neodymium-doped yttrium aluminum garnet and argon lasers were used to unclog the lumen and retract the iris tissue from the PMS. Following the procedures, the lumen was free from the iris tissue, and the IOP was normalized to 10 mmHg. His target IOP was achieved without any antiglaucoma medication to date.

In contrast to surgical repositioning or revision, laser therapy can be done in an outpatient setting. It is an effective and less invasive method of removing or avoiding iris tissue from obstructing the lumen of the PMS. Laser therapy offers an effective and safe option for managing PMS obstruction while maintaining shunt function with minimal to no complications.

## Introduction

Glaucoma is the second leading cause of irreversible blindness worldwide, with open-angle glaucoma being the most common subtype [1]. A systematic review by Tham et al. projects that the global prevalence of glaucoma will rise to 111.8 million by 2040 [2]. The progression of glaucoma can be delayed by lowering the intraocular pressure (IOP), as it remains the only modifiable risk factor. Various treatment options are available to achieve the target IOP, including medical therapy, laser treatments, and surgical interventions. The Preserflo MicroShunt (PMS) is a recently developed bleb-forming implant introduced by Santen Pharmaceutical Co., Ltd. in 2018. It falls under minimally invasive glaucoma surgery (MIGS) and has demonstrated effective IOP-lowering capabilities, particularly in moderate to advanced glaucoma cases [3].

The PMS is an ab externo glaucoma drainage device designed to reduce sustained IOP. It measures 8.5 mm in length, with an external diameter of 350 µm and a lumen diameter of 70 µm. This configuration allows for controlled aqueous humor outflow from the anterior chamber to the sub-Tenon’s space, facilitating the formation of a posterior filtering bleb. The posterior location of the bleb may reduce the risk of bleb-related complications, often associated with more anteriorly placed blebs, such as blebitis and endophthalmitis. Although generally considered safe, reported complications include transient hypotony, choroidal detachment, device exposure, shunt obstruction, and, in rare instances, malignant glaucoma. Furthermore, the PMS implantation procedure is less invasive than conventional filtration surgery and is typically associated with a more favorable and rapid postoperative recovery [3,4].

This report presents a case of PMS lumen obstruction caused by adjacent iris tissue due to shunt rotation, successfully managed with laser therapy without requiring implant repositioning. As the PMS is a relatively new addition to glaucoma management in our region, we hope this case serves as a valuable learning experience for clinicians encountering similar complications.

This article was previously presented as an e-poster at the International Virtual Medical Research Symposium 2023 on December 7th and 8th, 2023.

## Case presentation

**Figure 1:**
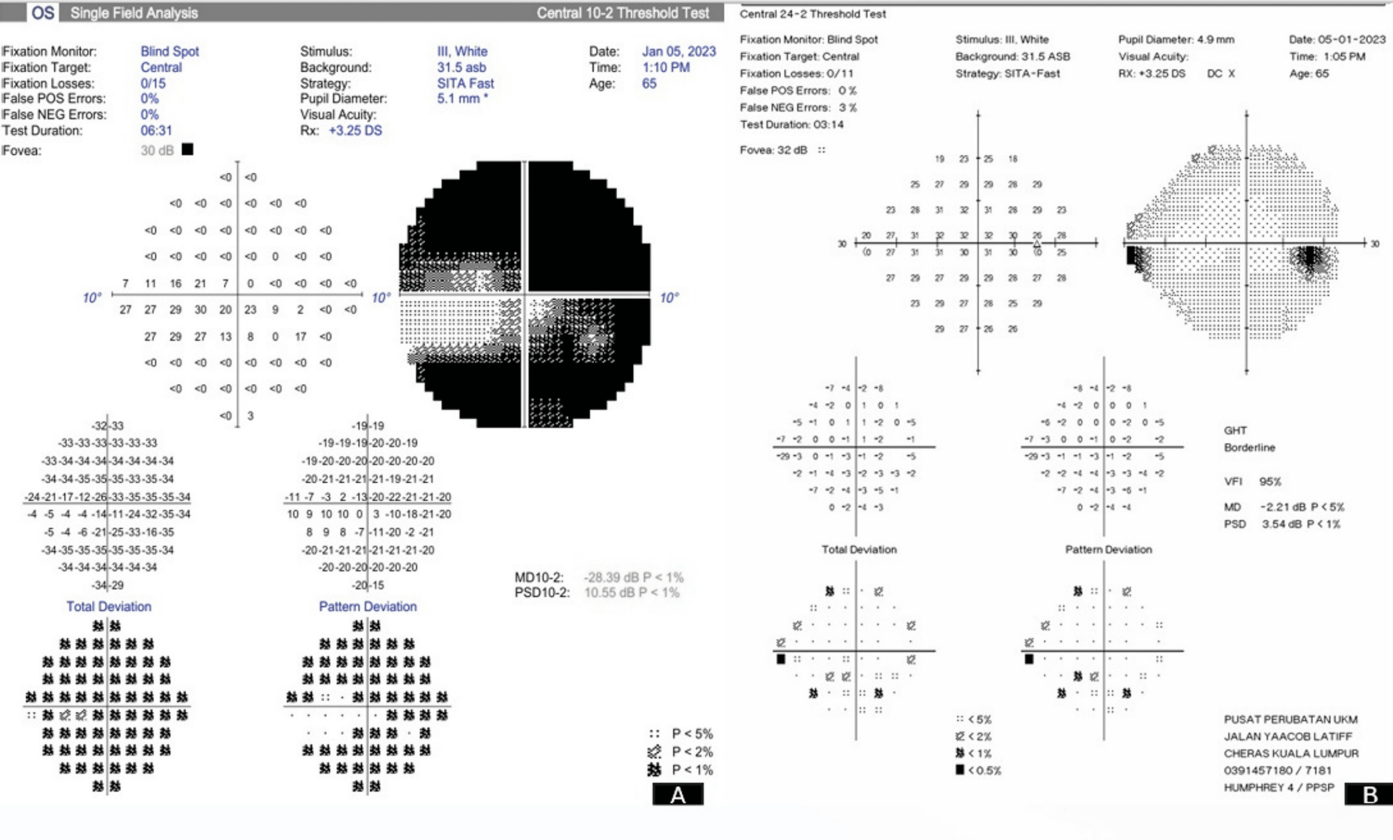
Humphrey visual field 24-2 (A) showing severe tunnel visual field with mean deviation of -28.39 dB over the left eye and (B) nasal step defect with mean deviation of -2.21 dB over the right eye

A 65-year-old gentleman with underlying hypertension, dyslipidemia, and ischemic heart disease was under ophthalmology follow-up for bilateral (OU) primary open-angle glaucoma. He was on maximal medical therapy, which included Gutt Latanoprost 0.005% nightly, Gutt Timolol 0.5% twice daily, Gutt Brimonidine 0.15% twice daily, and Gutt Brinzolamide 1% twice daily. His baseline best corrected visual acuity was 6/9 OU. Based on Hodapp-Parrish-Anderson criteria, his right eye was at an early stage, with a mean deviation of -2.99 dB, while his left eye was at a severe stage, with a mean deviation of -28.39 dB (Figure 1).

**Figure 2:**
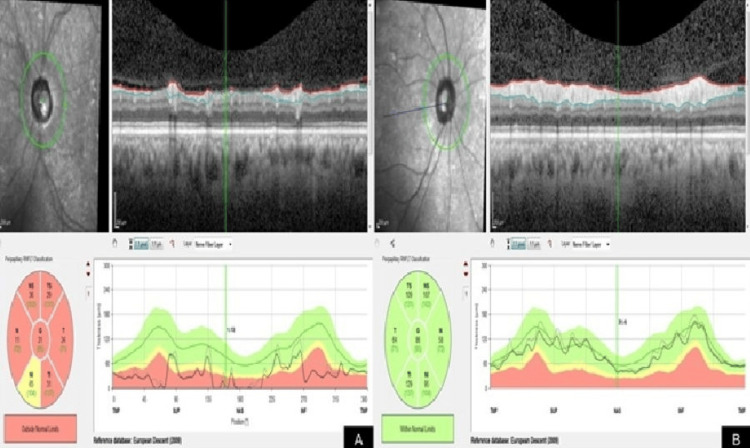
Peripapillary RNFL thickness analysis by spectral-domain optical coherence tomography. (A) Left eye showing generalized RNFL thinning, involving all the quadrants (red and yellow zones). (B) Right eye with RNFL measurements within normal limits across all quadrants RNFL: retinal nerve fiber layer

Spectral-domain optical coherence tomography of the peripapillary retinal nerve fiber layer (RNFL) demonstrated significant thinning in the left eye, involving all the quadrants. The RNFL thickness values in these regions fell below the lower limit of the normative database, as illustrated by the red and yellow sectors on the circular thickness map and the TSNIT graph. In contrast, the right eye showed RNFL measurements within normal limits across all quadrants (Figure 2).

Despite being on maximal antiglaucoma medication, the IOP of the left eye remained suboptimal, ranging from 25 mmHg to 32 mmHg measured with the Goldmann applanation tonometer. His right eye IOP ranged from 15 to 20 mmHg while on four antiglaucoma medications. Given the inadequate IOP control in the left eye, the patient was counseled and subsequently agreed to undergo PMS implantation augmented with mitomycin C 0.04%. His surgery was uneventful. Prior to conjunctival closure, the PMS was well-positioned, with its lumen facing anteriorly, and a steady aqueous flow was observed at the distal end of the PMS. On postoperative day one, his bleb was formed with an IOP of 8 mmHg. However, the lumen of the PMS had rotated nasally.

**Figure 3:**
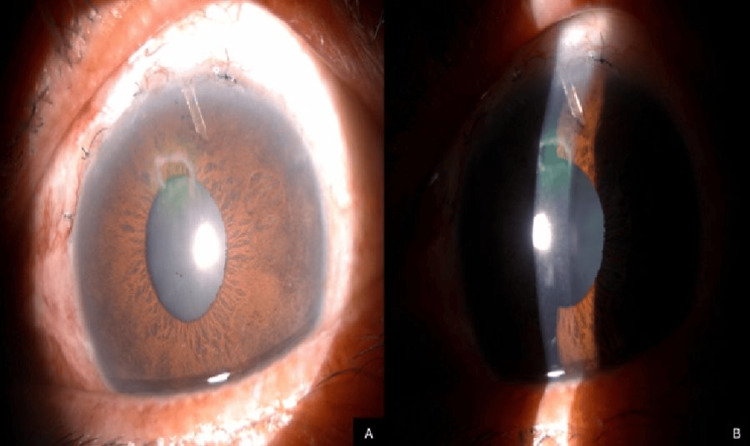
Anterior segment photos showing (A) the PMS implant, which was rotated nasally, causing the lumen to be partially obstructed by the adjacent iris tissue, and (B) worsening of the obstruction was noted when illumination of the slit lamp microscopy was reduced, causing the pupil to further dilate, iris tissue to heap up, and occlude the lumen completely PMS: Preserflo MicroShunt

At one week postoperatively, his IOP spiked to 30 mmHg, and anterior segment examination revealed that the lumen of the PMS was obstructed by adjacent iris tissue. The obstruction worsened in dim lighting, as pupil dilation allowed more iris tissue to clog the lumen (Figure 3).

Topical pilocarpine 2% was administered to relieve the obstruction through miosis. However, despite these measures, the iris-induced occlusion of the PMS persisted.

**Figure 4:**
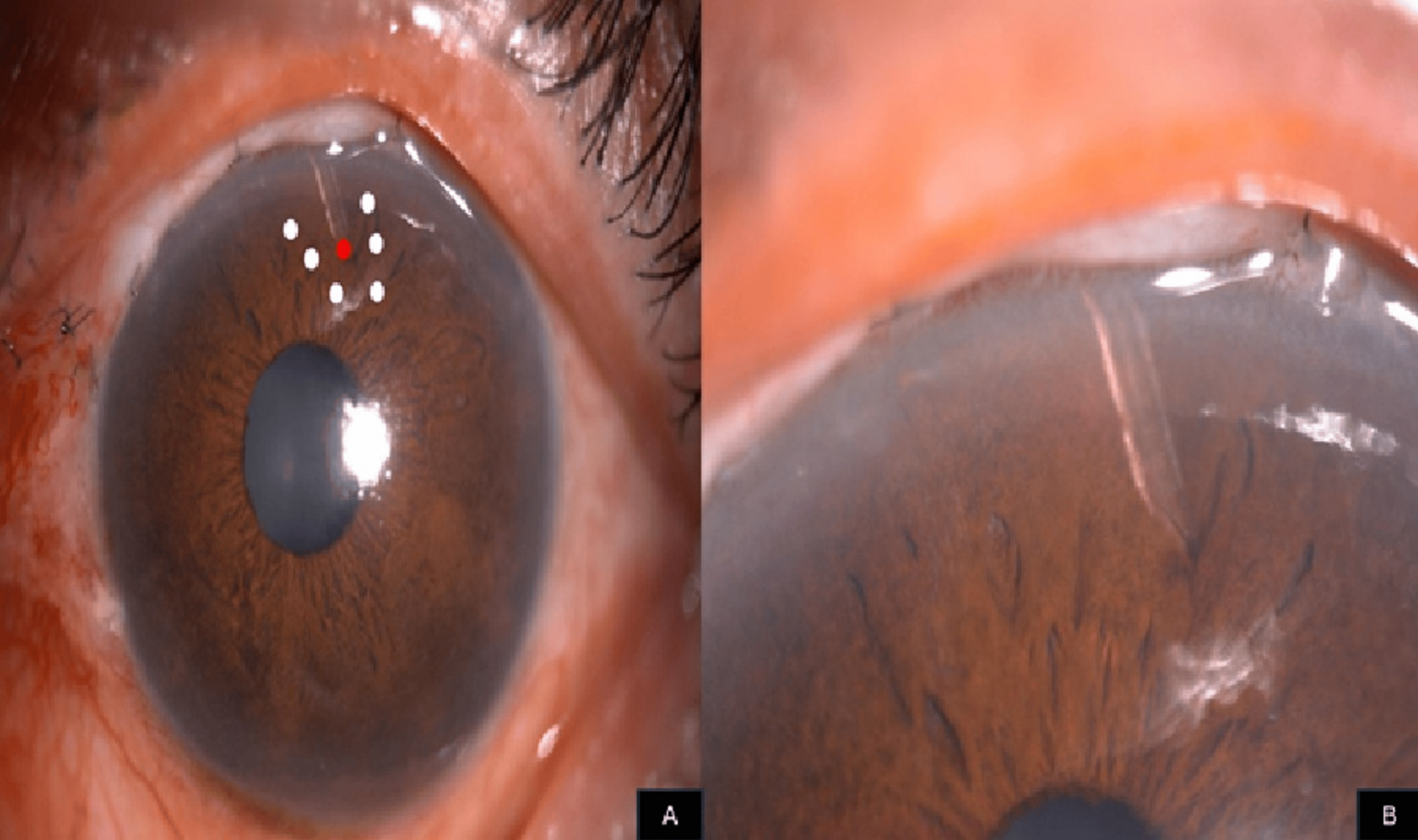
Anterior segment photos showing (A) the lumen of PMS, which was free from the iris tissue following Nd:YAG laser over the lumen (red dot) and argon laser over the surrounding iris tissue in a U-shaped pattern (white dots), with (B) the magnifying view showing a patent lumen of PMS and the surrounding flattened iris tissue post-procedure PMS: Preserflo MicroShunt, Nd:YAG: neodymium-doped yttrium aluminum garnet

Treatment options were discussed, and a neodymium-doped yttrium aluminum garnet (Nd:YAG) laser was performed with the setting of two shots x 1.0 mJ to remove the iris incarceration. Additionally, argon laser iridoplasty was performed (10 shots x 200 microns x 200 mW x 200 ms) in a U-shaped pattern around the lumen to contract the heaped-up iris tissue and retract it from the PMS. Following the procedure, the lumen was free of iris tissue, the surrounding iris was flat, and flow through the tube was re-established (Figure 4).

His IOP normalized to 10 mmHg. He continued to be medication-free with optimal IOP control in his left eye.

## Discussion

The major risk factor for glaucoma is increased IOP. Thus, the treatment of glaucoma primarily focuses on reducing IOP. If the target IOP is not achieved or disease progression occurs despite maximal medical therapy, laser therapy and filtering surgery are the next options [1]. MIGS has recently gained popularity due to its relatively low invasiveness and effective IOP reduction. The PMS is one of the MIGS procedures that has an effective IOP reduction in patients with open-angle glaucoma, particularly in moderate and advanced stages. The PMS is a minimally invasive bleb-forming implant that drains the aqueous humor from the anterior chamber to a fornix-based subconjunctival/Tenon’s flap. It bypasses the trabecular meshwork, which is assumed to have the highest resistance for drainage of aqueous humor. The lumen of the tube is designed to be narrow enough to prevent overfiltration, yet wide enough to resist obstruction by inflammatory cells or pigments [4].

Adverse events associated with PMS implantation include transient postoperative hypotony, shallowing of the anterior chamber, hyphaema, transient choroidal detachment, obstructed shunt, and bleb fibrosis [1]. Most complications are mild in severity and can be managed successfully without any sight-threatening complications [5].

There have been a few reports of PMS lumen obstruction with iris tissue following implantation and its successful treatment with Nd:YAG laser or surgical intervention [6-8]. Trigaux et al. reported a case of successful removal of the obstructing iris fibers with retinal scissors and forceps [8]. Iwasaki et al. reported a case of tube obstruction by iris capture, triggered by intraocular lens (IOL) displacement after PMS implantation and its successful surgical management without PMS implant reposition. The IOL was repositioned, and the iris was removed from the tube lumen using a Sinskey hook [7].

A similar iris obstruction has also been reported in other MIGS implants, such as the XEN Gel Stent, which has a smaller lumen than the PMS. In this case, the obstruction was successfully treated with a combination of Nd:YAG laser and argon laser iridoplasty, following failed revision surgery for recurrent iris incarceration [9]. There is no consensus on managing glaucoma drainage device (GDD) obstruction; however, it is widely managed with either surgical intervention or laser therapy. Surgical removal of the obstruction using forceps or repositioning of the PMS has been reported to unclog the obstruction by vitreous or blood. In cases of iris incarceration, Nd:YAG or argon lasers have been previously reported to be successful in restoring the function of the tube [8].

Risk factors for iris obstruction of the PMS include shallowing of the anterior chamber, transient hypotony, and tube contact with the iris [6]. Our patient had some degree of iris-lumen contact, especially when the iris is dilated in dim surroundings. We propose that rotation of the PMS and nasal displacement of the lumen of the PMS may have occurred due to an unintentionally enlarged sclerotomy wound before the implantation, causing the fin of the PMS to rotate postoperatively, as it was one of the pioneer implantations in our center. In our case, after using the Nd:YAG laser, we complement it with the argon laser over the surrounding iris tissue to flatten it. An argon laser surrounding the lumen was used to level out the iris and avoid the recurrence of this adverse event. While laser therapy for lumen obstruction carries potential risks such as pigment dispersion syndrome, IOP fluctuations, and anterior segment inflammation, our patient experienced only mild post-laser inflammation, which responded well to a short course of topical corticosteroids. No evidence of pigment dispersion or shunt damage was observed during follow-up. Laser therapy was chosen over surgical repositioning as it is less invasive, faster, and easier to perform in an outpatient setting. Other alternatives were either surgical intervention by surgically removing the obstruction or reinsertion of the shunt.

## Conclusions

This case highlights a complication of PMS lumen obstruction by adjacent iris tissue due to shunt rotation, which was successfully managed using a combination of Nd:YAG laser and argon laser iridoplasty. Given the increasing adoption of MIGS, recognizing and addressing such complications is essential to optimizing patient outcomes. Laser therapy proved to be a minimally invasive, effective, and outpatient-friendly alternative to surgical intervention, restoring aqueous flow and achieving IOP control without the need for implant repositioning.
